# The P300/XBP1s/Herpud1 axis promotes macrophage M2 polarization and the development of choroidal neovascularization

**DOI:** 10.1111/jcmm.16673

**Published:** 2021-05-31

**Authors:** Wendie Li, Ying Wang, Linling Zhu, Shu Du, Jinghai Mao, Yanyan Wang, Sangsang Wang, Qingyun Bo, Yuanyuan Tu, QuanYong Yi

**Affiliations:** ^1^ Department of Ophthalmology Ningbo Eye Hospital Ningbo China; ^2^ Department of Ophthalmology Suzhou Municipal Hospital The Affiliated Suzhou Hospital of Nanjing Medical University Suzhou China; ^3^ Department of Ophthalmology Lixiang Eye Hospital of Soochow University Suzhou China

**Keywords:** choroidal neovascularization, Herpud1, macrophage polarization, p300, XBP1s

## Abstract

Neovascular age‐related macular degeneration (AMD), which is characterized by choroidal neovascularization (CNV), leads to vision loss. M2 macrophages produce vascular endothelial growth factor (VEGF), which aggravates CNV formation. The histone acetyltransferase p300 enhances the stability of spliced X‐box binding protein 1 (XBP1s) and promotes the transcriptional activity of the XBP1s target gene homocysteine inducible endoplasmic reticulum protein with ubiquitin‐like domain 1 (Herpud1). Herpud1 promotes the M2 polarization of macrophages. This study aimed to explore the roles of the p300/XBP1s/Herpud1 axis in the polarization of macrophages and the pathogenesis of CNV. Hypoxia‐induced p300 interacted with XBP1s to acetylate XBP1s in RAW264.7 cells. Additionally, hypoxia‐induced p300 enhanced the XBP‐1s‐mediated unfolded protein response (UPR), alleviated the proteasome‐dependent degradation of XBP1s and enhanced the transcriptional activity of XBP1s for Herpud1. The hypoxia‐induced p300/XBP1s/Herpud1 axis facilitated RAW264.7 cell M2 polarization. Knockdown of the p300/XBP1s/Herpud1 axis in RAW264.7 cells inhibited the proliferation, migration and tube formation of mouse choroidal endothelial cells (MCECs). The p300/XBP1s/Herpud1 axis increased in infiltrating M2‐type macrophages in mouse laser‐induced CNV lesions. Blockade of the p300/XBP1s/Herpud1 axis inhibited macrophage M2 polarization and alleviated CNV lesions. Our study demonstrated that the p300/XBP1s/Herpud1 axis in infiltrating macrophages increased the M2 polarization of macrophages and the development of CNV.

## 1 INTRODUCTION

Age‐related macular degeneration (AMD) is divided into early‐stage, intermediate‐stage and advanced‐stage AMD according to its course. Among them, advanced‐stage AMD can be classified into non‐neovascular and neovascular AMD.^1^, ^2^ Neovascular AMD is characterized by choroidal neovascularization (CNV), in which abnormal neovasculature results in intraretinal and subretinal haemorrhage and macular oedema, finally leading to severe subretinal fibrosis, with 90% of AMD patients experiencing vision loss.^3^

The mechanisms of CNV have remained unknown until now. However, accumulating study has revealed that genetic and environmental factors such as ageing, diet, inflammation, and oxidative stress have been linked to CNV.^4^ It has been reported that immune response and inflammation contribute to CNV pathogenesis.^5^ Macrophages are the major immune cell type that infiltrates CNV lesions.^6^ According to distinct pathways of their activation, macrophages can be broadly classified into two subsets, M1 (or classically activated macrophages) and M2 (or alternatively activated macrophages, AAMs). M1 macrophages produce the molecules interleukin‐6 (IL‐6), inducible nitric oxide synthase (iNOS) and T‐lymphocyte activation antigen CD86 (CD86), while M2 macrophages produce arginase 1 (Arg1), chitinase‐3‐like protein 3 (Ym1) and mannose receptor C‐Type 1 (CD206); these proteins are used as M1 and M2 macrophage markers, respectively. Additionally, some studies have revealed that the infiltration of CNV lesions by M2 macrophages promotes the progression of CNV by M2 macrophage‐mediated secretion of vascular endothelial growth factor (VEGF).^7^, ^8^

The endoplasmic reticulum (ER), which is found in every eukaryotic cell, facilitates a wide variety of cellular functions, such as protein folding, free calcium storage and lipid/sterol synthesis.^9^ The ER has its own regulatory mechanism called the unfolded protein response (UPR), which is activated as an effort to regain ER homeostasis and to restrict further injury to the cell.^10^ The UPR regulates macrophage polarization, facilitating the M1 to M2 switch. For example, homocysteine inducible endoplasmic reticulum protein with ubiquitin‐like domain 1 (Herpud1) is upregulated in interleukin‐4 (IL‐4)‐treated M2 macrophages, and its expression pattern is similar to that of macrophage polarization markers, such as Arg1 and mannose receptor (Mrc1). Inhibition of Herpud1 with specifically targeted short hairpin RNA (shRNA) decreases the expression of these markers at the mRNA and protein levels in IL‐4‐treated and untreated M2 macrophages. Furthermore, IL‐4 treatment promotes M2 macrophage migration and polarization, but this effect is weakened by Herpud1 depletion.^11^ Additionally, Herpud1 has been reported to increase the level of amyloid β (Aβ), a component of drusen deposits underlying the retinal pigment epithelium (RPE) layer during CNV.^12^

Herpud1 is transcribed by the transcription factor X‐box binding protein 1 (XBP1), as XBP1 is shown to bind very strongly to the promoter of Herpud1 at the unfolded protein response element (UPRE) sequence TGACGTGG (10291‐19298), located at −54 to −47 relative to the beginning of the human Herpud1 promoter.^13^ XBP1 acts as an effector central to the UPR. XBP1 expression has been found to be induced in macrophages alternatively activated by IL‐4/IL‐13 (M2 macrophages). Meanwhile, XBP1 inhibition substantially reduces the IL‐6‐mediated hyperpolarization of macrophages, suggesting that XBP1 contributes to the M2 polarization of macrophages.^14^ Additionally, XBP1s is a target of acetylation mediated by histone acetyltransferase p300 (p300), which increases acetylation and protein stability of XBP1s, and enhances the transcriptional activity of XBP1s.^15^

XBP1 is activated by the ER stress sensor inositol‐requiring enzyme 1α (IRE1α), a transmembrane protein kinase in the ER that oligomerizes upon the accumulation of unfolded proteins in the ER lumen. Oligomerization and auto‐transphosphorylation activate the RNase function of IRE1α, which mediates the unconventional splicing of XBP1 mRNA in the cytosol.^16^ Removal of a 26‐nt intron comprising an N‐terminal basic leucine zipper (bZIP) domain followed by a C‐terminal transcription activation domain from XBP1 mRNA leads to a frameshift and expression of the transcription factor XBP1s.

Here, we explored the role of the p300/XBP1s/Herpud1 axis in macrophage polarization during CNV development. Our results indicated that the activation of the p300/XBP1/Herpud1 axis inside macrophages promoted the M2 polarization of macrophages. Consequently, the presence of M2 macrophages enhanced the proliferation, migration and tube formation of choroidal endothelial cells, exacerbating the development of CNV. Our study further revealed the molecular mechanisms of macrophage polarization during CNV pathogenesis.

## 2 MATERIALS AND METHODS

### 2.1 Cell culture and treatment

RAW264.7 cells (#TIB‐71) and HEK293T cells (#CRL‐3216) purchased from American Type Culture Collection were cultured in Dulbecco's modified Eagle's medium (DMEM; #11960044, Gibco, USA) supplemented with 10% (v/v) foetal bovine serum (FBS; #10091, Gibco) and 1% penicillin/streptomycin (#15140122, Gibco) at 37℃ and 5% CO_2_ in a humidified incubator. The isolation and culture of mouse choroidal endothelial cells (MCECs) were performed as previously described.^17^ Cells cultured under 95% O_2_ and 5% CO_2_ conditions for 24 hours were used as the normal (normoxia) group, while cells cultured in 95% N_2_/5% CO_2_ for 24 hours were used as the hypoxia group. The p300 inhibitor L002 diluted in 0.1% DMSO was added to RAW2647 cells at a dose of 5 μmol/L for 24 hours. The same volume of 0.1% DMSO was added to the control RAW264.7 cells. The XBP1 agonist HLJ2 at 1 μmol/L was added to RAW264.7 cells and incubated for 24 hours, after which the RAW264.7 cells were subjected to hypoxia and administered STF‐083010, an inhibitor of XBP1 splicing, at 50 μmol/L for 24 hours.

### 2.2 Western blot

Cell or tissue lysates and prestained protein molecular weight markers (#26612, Pierce, USA) were separated by sodium dodecyl sulphate‐polyacrylamide gel electrophoresis (SDS‐PAGE), followed by transferring onto polyvinylidene fluoride (PVDF) membranes. The membranes were blocked in Tris‐buffered saline with 0.5% Tween 20 (TBST) containing 5% bovine serum albumin (BSA) and probed with primary antibodies overnight at 4℃. Then, the membranes were washed three times and incubated with the horseradish peroxidase (HRP)‐conjugated secondary antibodies goat‐anti‐rabbit IgG (#7074) and horse‐anti‐mouse IgG (#7076). The primary antibodies used in the study were anti‐p300 (#86377), anti‐p‐IRE1α (#PA1‐16927, Invitrogen, USA), anti‐IRE1α (#3294), anti‐XBP1s (#24868‐1‐AP, Proteintech, USA), anti‐acetylated lysine (Ac‐K; #9681), anti‐activating transcription factor 4 (ATF4; #11815), anti‐glucose regulated protein 94 (GRP94; #2104), anti‐binding immunoglobulin protein (BIP; #3183), anti‐C/EBP homologous protein (CHOP; #2895), anti‐ubiquitin (#3933), anti‐Herpud1 (ab150424, Abcam, USA), and anti‐GAPDH (#2118). Antibodies whose supplier is not stated were purchased from Cell Signalling Technology (USA). Immunoreactivity was visualized by enhanced chemiluminescence (ECL, #34577, Pierce, USA).

### 2.3 Co‐immunoprecipitation (co‐IP)

RAW264.7 cells were randomly assigned into normal control, hypoxia, hypoxia +0.1% DMSO (vehicle) and hypoxia +L002 (p300 inhibitor; 5 μmol/L for 24 hours) groups. The Catch and Release® v2.0 reversible immunoprecipitation system (#17‐500, Millipore, USA) was used for co‐IP. The cells were lysed, after which 2 μg/mL anti‐XBP1s (#40435) or anti‐p300 (#86377) antibodies purchased from Cell Signalling Technology were used to precipitate the proteins. Mouse anti‐rabbit IgG (#3678, Cell Signalling Technology; 1:1000) antibody was used as a negative control. The precipitated proteins were resolved by SDS‐PAGE and transferred to PVDF membranes. The membranes were incubated with primary antibodies against Ac‐K, XBP1s, p300 and GAPDH overnight at 4℃, followed by incubation with HRP‐conjugated goat‐anti‐rabbit IgG (31460, Invitrogen, USA; 1:1000), and the signals werer developed using the ECL method.

### 2.4 Cycloheximide (CHX) chase assay

HEK293T cells were randomly assigned to the pcDNA3.1‐p300 (#23252, Addgene, USA) + Flag‐XBP1s (#63678, Addgene), pcDNA3.1‐p300 + Flag‐XBP1s + L002, pcDNA3.1‐p300 + Flag‐XBP1s + MG132 and pcDNA3.1‐p300 + Flag‐XBP1s + L002 + MG132 groups and then treated with CHX (#S5397, Selleck, USA; 20 μg/mL) for 0, 15, 30, and 60 minutes. XBP1s protein levels were determined by Western blot.

### 2.5 Ubiquitination assay

HEK293T cells were transfected with ubiquitin (#U‐100H, R&D Systems, USA) and the pcDNA3.1‐p300 and Flag‐XBP1s plasmids using Lipofectamine 3000 (#L3000008, Thermo Fisher Scientific). After 12 hours of transfection, 5 μmol/L L002 was added to the medium for 24 hours. After 36 hours of transfection, 20 μmol/L MG132 (#S2619, Selleck Chemicals, USA) was added to the medium for 4 hours, followed by protein extraction. Cell lysates were co‐IP by overnight incubation with anti‐XBP1s antibody at 4℃. The eluted proteins were determined by Western blot using anti‐ubiquitin (#ab7780, Abcam), anti‐XBP1s, anti‐p300 and anti‐GAPDH antibodies.

### 2.6 Transient DNA transfection and luciferase reporter assay

The 3’‐UTR of Herpud1 and 3 mutant sequences were amplified by PCR with primers containing Mlu I and Hind III restriction sites on each 5’ or 3’ strand. The PCR products were inserted into the Mlu I and Hind III sites of the pMIR‐REPORT luciferase vector and verified through DNA sequencing.^18^ Wild‐type plasmid contained the 3’‐UTR of Herpud1 with the UPRE sequence 5’‐TGACGTGG‐3’, while mutant‐type plasmid contained the sequence 5’‐AATCACAG‐3’. HEK293T cells were co‐transfected with 200 ng of pcDNA3.1‐p300, Flag‐XBP1s, and a reporter plasmid and 10 ng of pRL‐CMV vector (internal control; #E2261, Promega, USA). After transfection, the medium was replaced with serum‐free DMEM, and the cells were cultured for 16 hours. Firefly and Renilla luciferase assays were performed using the Dual‐Luciferase Reporter Assay System (#E1910, Promega). Firefly luciferase activity (relative light units) was determined using an Infinite 200 multiplate reader (Tecan, USA) and normalized to Renilla luciferase activity.

### 2.7 Quantitative reverse transcription‐polymerase chain reaction (qRT‐PCR)

mRNA extracted from RAW264.7 cells was reverse transcribed in a 20 µL final volume from 400 ng of total RNA using a TaKaRa PrimeScript II First‐strand Complementary DNA (cDNA) Synthesis Kit (#D6210A; TaKaRa, Japan). mRNA levels of the M1‐type macrophage markers IL‐6, iNOS and CD86 and the M2‐type macrophage markers Arg1, Ym1 and CD206 were detected with the following forward and reverse primers: IL‐6 (5′‐AACGATGATGCACTTGCAGA‐3′ and 5′‐GTGACTCCAGCTTATCTCTTGGT‐3′); iNOS (5′‐TCTAGTGAAGCAAAGCCCAACA‐3′ and 5′‐TCCAGAGGGGTAGGCTTGTC‐3′); CD86 (5′‐CAGCACGGACTTGAACAACC‐3′ and 5′‐TGTGCCCAAATAGTGCTCGT‐3′); Arg1 (5′CGGGAGGGTAACCATAAGCC‐3′ and 5′‐CTTGGGAGGAGAAGGCGTTT‐3′); Ym1 (5′‐GGGCCCTTATTGAGAGGAGC‐3′ and 5′‐CCAGCTGGTACAGCAGACAA‐3′); CD206 (5′‐AGTCAGAACAGACTGCGTGG‐3′ and 5′‐CCAGAGGGATCGCCTGTTTT‐3′) and GAPDH (5′‐AAGAGGGATGCTGCCCTTAC‐3′ and 5′‐TACGGCCAAATCCGTTCACA‐3′). GAPDH was used as the loading control. Reactions were performed in 20 µL volumes containing 2× SYBR Premix Ex Taq (#RR820B, TaKaRa), forward PCR primer (10 μmol/L), reverse PCR primer (10 μmol/L), cDNA template, and double‐distilled water using a Bio‐Rad CFX96 real‐time PCR system. Data were collected and calculated using Bio‐Rad CFX Manager 1.6 software. RNA expression was calculated based on a relative standard curve with the 2^‐ΔΔCt^ method.

### 2.8 5‐Ethynyl‐2´‐deoxyuridine (EdU) incorporation assay

MCECs were cultured in 24 well plates for 24 hours and then treated with 50 μmol/L EdU‐555 (#17‐10526, Millipore, USA) for 2 hours at 37℃. The cells were fixed in 4% paraformaldehyde (PFA) for 10 minutes and permeabilized with 0.5% Triton X‐100 for 10 minutes at room temperature. Subsequently, the cell nuclei were stained with DAPI (#268298, Sigma‐Aldrich, USA) and visualized under a fluorescence microscope (Olympus, Japan). The ratio of proliferative cells was calculated as the ratio of EdU‐positive cells to DAPI‐positive cells.

### 2.9 Transwell migration assay

A total of 1 × 10^4^ MCECs were added to the upper chamber of Transwell cell culture inserts (#CLS3464, Corning, USA) with 200 μL of serum‐free medium, and the bottom chamber was filled with complete medium. After incubation for 48 hours, the cells on the lower surface were fixed with 95% ethanol and then stained with 0.1% crystal violet (#C0121‐100 mL, Beyotime, China). Five random fields from each well were photographed and analysed using an Olympus microscope and ImageJ software (National Institutes of Health, USA), respectively.

### 2.10 Tube formation assay

Tissue culture plates (96‐well) were coated with 400 μL of growth factor‐reduced basement membrane matrix (Matrigel; #356252, Corning, USA). MCECs were seeded at a density of 1 × 10^6^ cells/well and treated with serum‐free DMEM containing recombinant mouse VEGF (#493‐MV, R&D Systems) at a dose of 30 ng/mL for 17 hours at 37℃. Capillary‐like tube structures formed by MCECs on the Matrigel were photographed with a digital camera (DP71, Olympus, Japan). Tube formation was analysed by quantifying the tube length of the capillary‐like structures per visual field.

### 2.11 Mouse laser‐induced CNV model

All animal studies were approved by the Committee on the Ethics of Animal Experiments of Soochow University. Male C57BL/6J mice aged 6‐8 weeks were subjected to 532 nm argon laser‐induced photocoagulation in both eyes. The mice had been anaesthetized with pentobarbital sodium (#4579, Tocris, USA; 2% w/v). After the pupils were dilated with tropicamide, four laser photocoagulation spots in the posterior pole of the retina were created through the dilated pupil with a power of 150 mW, spot diameter of 50 µm and duration of 100 ms Laser‐induced spots were located approximately 2.5‐3 disc diameters from the optic nerve head, avoiding the main vessels. A white, gaseous bubble formed at each spot, indicating the rupture of Bruch's membrane, subsequently leading to CNV. Two mice were excluded due to haemorrhage. The mice were woken up under a heat lamp after laser photocoagulation.

### 2.12 Animal treatment

The mice were randomly assigned into the following groups: normal, CNV 7 d, CNV 7 d +0.1% DMSO (administered in a 5‐μl volume by intraperitoneal injection from day 1 to day 6), CNV 7 d + L002 (administered at 20 μg/kg/day body weight by intraperitoneal injection from day 1 to day 6), CNV 7 d + STF‐083010 (administered at 50 mg/kg/day body weight by intraperitoneal injection from day 1 to day 6) and CNV 7 d + Herpud1 siRNA (administered at 3 μM at day 5 by intravitreal injection).

### 2.13 Tissue immunofluorescence

On day 7, following euthanasia, the mouse eyes were enucleated and immersion‐fixed in 10% PFA for 2 hours. After fixation, the eyes were embedded in Tissue‐Tek® OCT compound (#25608‐930, Finetek, Japan) and vertically cross‐sectioned through the centre of the cornea and optic nerve on a cryostat. Slides with a thickness of 5 µm were incubated with anti‐Arg‐1 (#66129‐1‐Ig, Proteintech, USA)/anti‐p300, anti‐Arg‐1/anti‐XBP1s, anti‐Arg‐1/anti‐Herpud1, and anti‐Arg‐1/Alexa Fluor™ 488‐anti‐isolectin B4 (IB4; I21414, Invitrogen) antibodies overnight at 4℃. After washing, the slides were incubated with phycoerythrin (PE)‐goat‐anti‐mouse IgG (#ab97024, Abcam) and fluorescein isothiocyanate (FITC)‐goat‐anti‐rabbit IgG (#F‐2765, Invitrogen, USA). The nuclei were counterstained with DAPI. Mounted slides were imaged with an Olympus microscope.

### 2.14 Fundus fluorescein angiography (FFA) and indocyanine green angiography (ICGA)

Mice were anaesthetized and prepared as described above on day 7 after laser‐induced CNV. Fluorescein sodium (0.05 mL, 100 mg/mL; #2119436, Akorn, USA) or indocyanine green (#1340009, Sigma‐Aldrich) was injected into the mouse tail vein. The fundus was imaged using a Micron3 fundus camera (Phoenix Technology Group, USA). After imaging, the mice were given erythromycin ophthalmic ointment (#2807667, Akorn) and placed in a heated recovery cage. The leakage area was assessed by a reviewer blinded to the treatment group and genotype by using a freehand tool to outline the area of leakage, after which the region of interest (ROI) manager tool in ImageJ was used to calculate the area in pixels. Leakage was graded by two blinded reviewers on a scale of 0, 1, 2a and 2b as previously described.^19^

### 2.15 Statistical analyses

Data are presented as the mean ± SEM. Statistical analyses were performed using two‐tailed, unpaired, Student's *t*‐test or one‐way ANOVA, followed by Tukey post‐test analysis as appropriate using GraphPad Prism version 5 (GraphPad Software Inc, USA). A *P* value of less than .05 was used to indicate statistical significance.

## 3 RESULTS

### 3.1 Hypoxia‐induced p300 interacted with XBP1s to acetylate XBP1s in RAW264.7 cells

**FIGURE 1:**
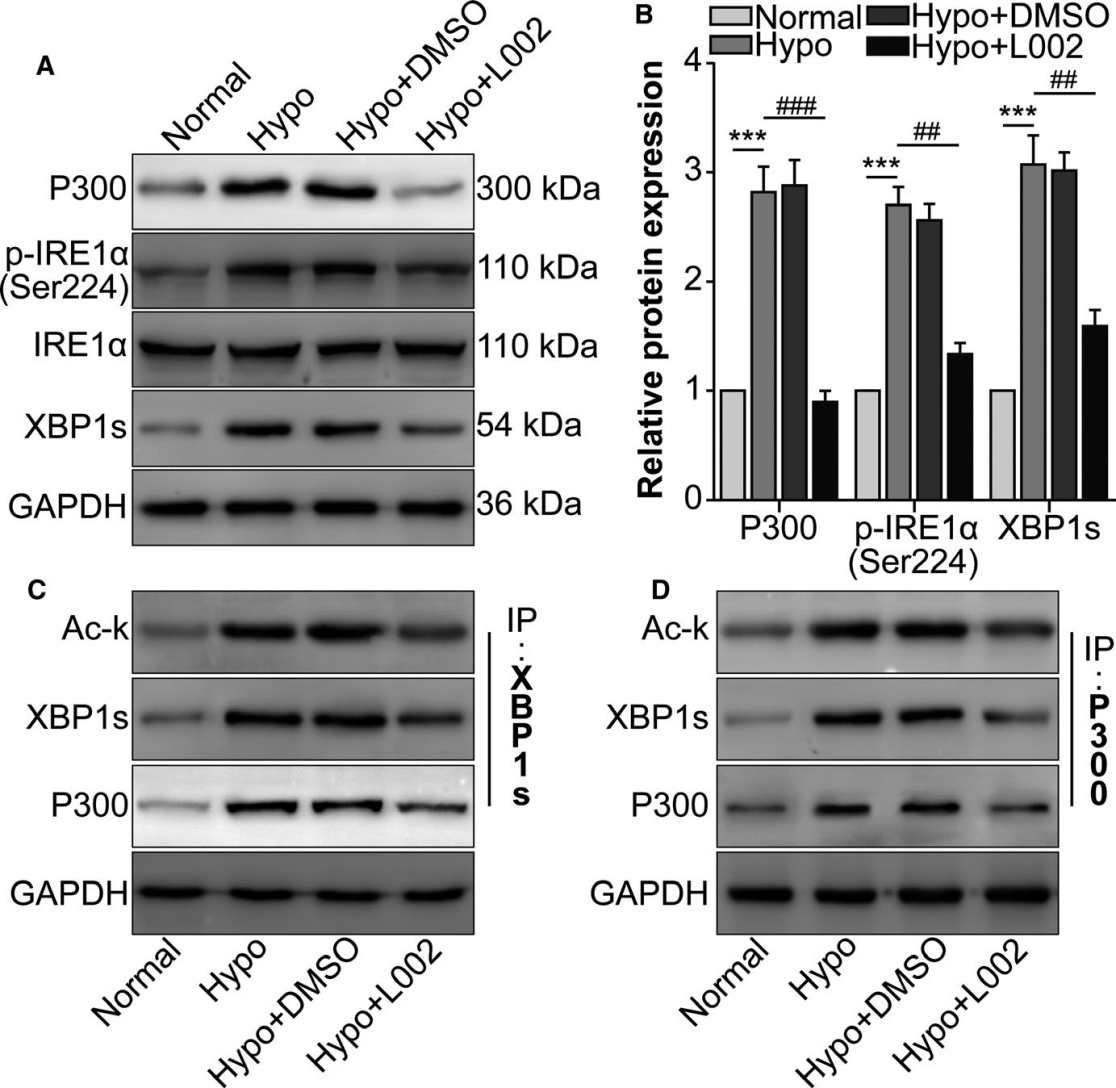
Hypoxia‐induced p300 interacted with XBP1s to acetylate XBP1s in RAW264.7 cells. RAW264.7 cells were assigned to normal, hypoxia, hypoxia +0.1% DMSO (vehicle) and hypoxia +L002 groups. A, Western blot was performed to detect p300, p‐IRE1α (Ser724), IRE1α and XBP1s protein levels. B, The relative protein levels of p300, p‐IRE1α and XBP1s were analysed. ^***^ *P* <.005 vs the normal group; ^###^ *P* <.005 vs the hypoxia group. C, RAW264.7 cell lysates were immunoprecipitated with anti‐XBP1s antibody and immunoblotted with anti‐Ac‐K and anti‐p300 antibodies. D, RAW264.7 cell lysates were immunoprecipitated with anti‐p300 antibody and immunoblotted with anti‐Ac‐K and anti‐XBP1s antibodies. n = 3/group

First, p300, p‐IRE1α and XBP1s in RAW264.7 cells induced by hypoxia were found to be decreased by the p300 inhibitor L002 (Figure 1A,B). Under hypoxic conditions, the interaction between p300 and XBP1 and the acetylation of XBP1 increased compared to those in the normal group, while p300 inhibition reduced the interaction between p300 and XBP1s and acetylation of XBP1 (Figure 1C,D). These data showed that hypoxia‐induced p300 interacted with XBP1s to acetylate XBP1s in RAW264.7 cells.

### 3.2 Hypoxia‐induced p300 enhanced XBP‐1s‐mediated UPR

**FIGURE 2:**
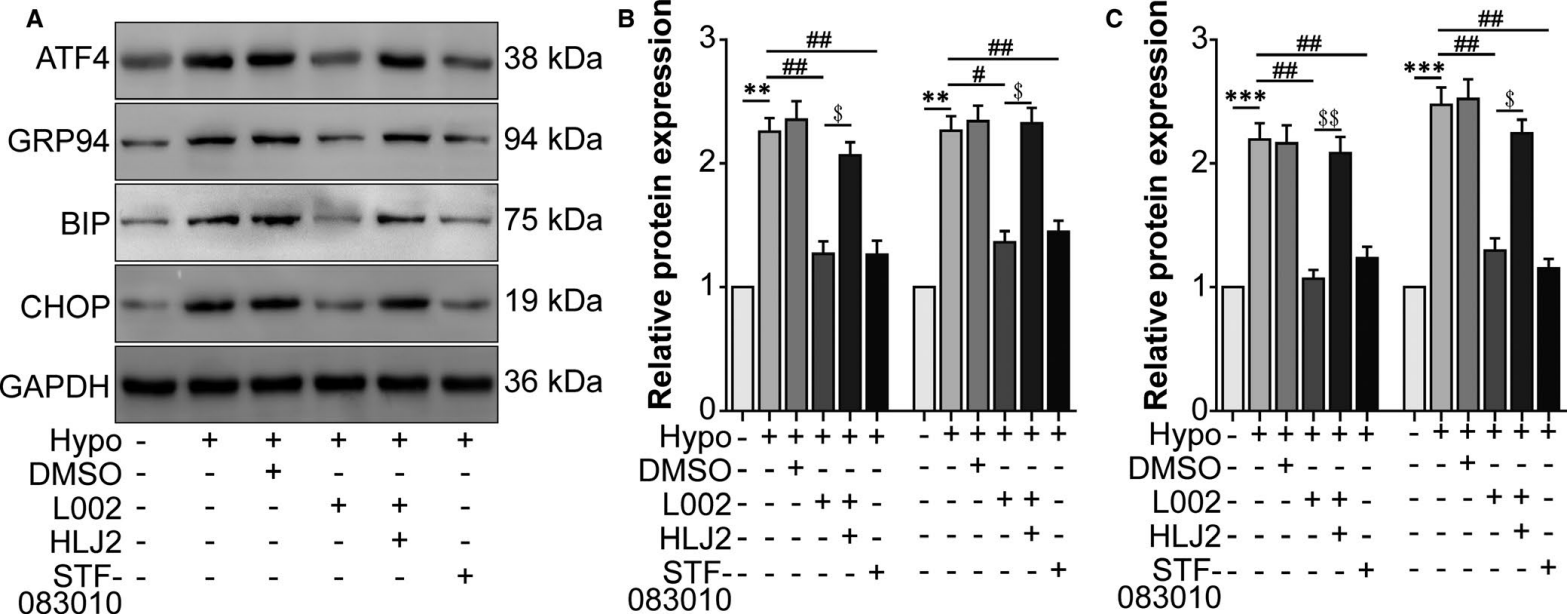
Hypoxia‐induced p300 enhanced the XBP‐1s‐mediated unfolded protein response (UPR). RAW264.7 cells were assigned to normal, hypoxia, hypoxia +0.1% DMSO, hypoxia +L002, hypoxia +L002 + HLJ2 and hypoxia +STF‐083010 groups. A, Western blot was performed to detect protein levels of the UPR‐associated molecules ATF4, GRP94, BIP and CHOP. B and C, The relative protein levels of each molecule were analysed. ^**^ *P* <.01, ^***^ *P* <.005 vs the normal group; ^#^ *P* <.05, ^##^ *P* <.01 vs the hypoxia group. ^$^ *P* <.05, ^$$^ *P* <.01 vs the hypoxia +L002 group. n = 3/group

Next, we identified the effect of p300 on XBP‐1s‐mediated UPR. Western blot showed that the UPR‐associated molecules ATF4, GRP94, BIP and CHOP increased in the hypoxia group compared to the normal group. L002 and the XBP1 splicing inhibitor STF‐083010 decreased the expression of these UPR‐associated molecules, while the XBP1 agonist HLJ2 reversed the effect of L002 (Figure 2A‐C). These results suggested that hypoxia‐induced p300 promoted the UPR via the activation of XBP1 in RAW264.7 cells.

### 3.3 Hypoxia‐induced p300 alleviated the proteasome‐dependent degradation of XBP1s and enhanced the transcriptional activity of XBP1s for Herpud1

**FIGURE 3:**
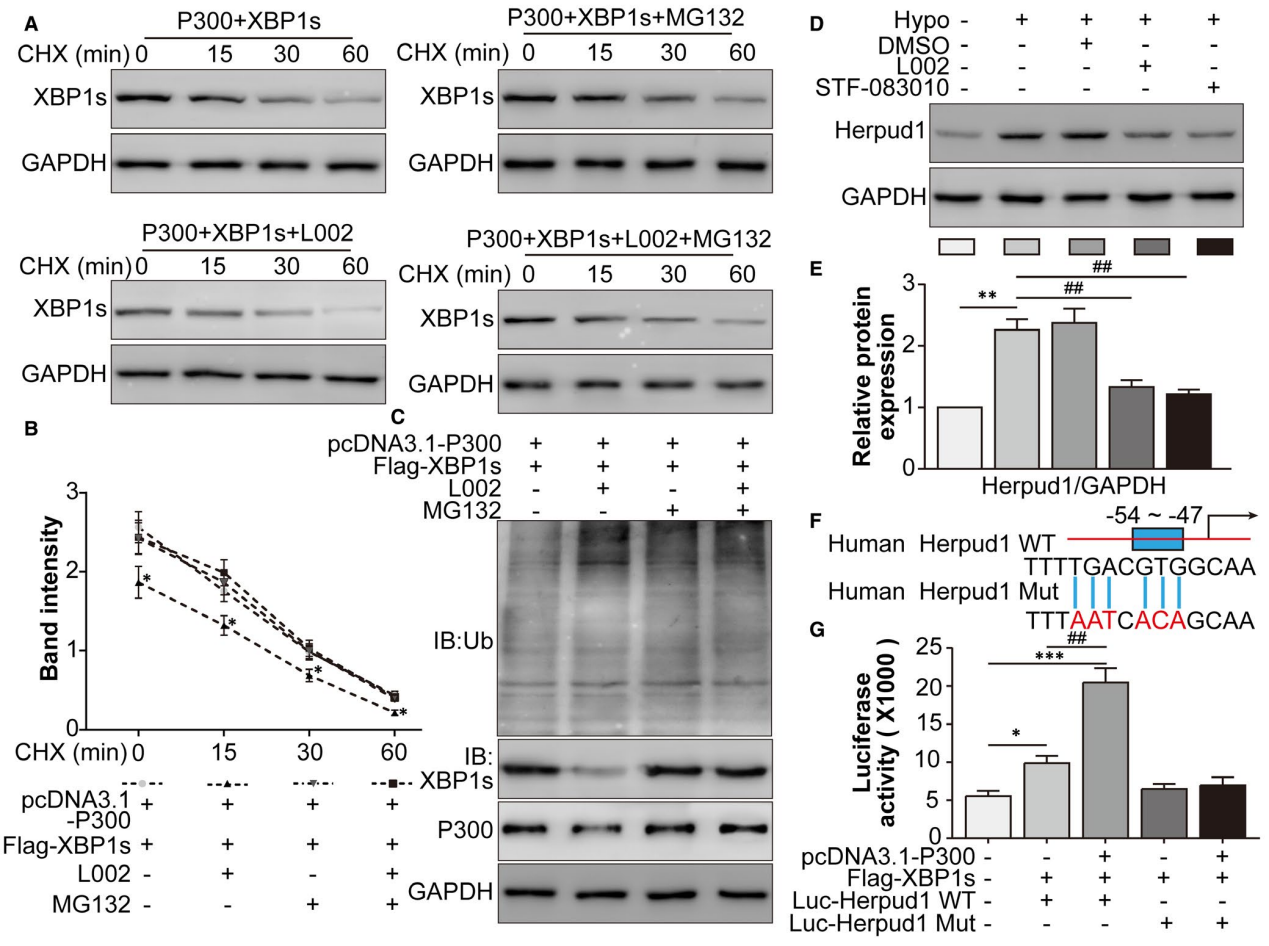
Hypoxia‐induced p300 alleviated the proteasome‐dependent degradation of XBP1s and enhanced the transcriptional activity of XBP1s for Herpud1. A, HEK293T cells were assigned to the pcDNA3.1‐p300 + Flag‐XBP1s, pcDNA3.1‐p300 + Flag‐XBP1s + L002, pcDNA3.1‐p300 + Flag‐XBP1s + MG132 and pcDNA3.1‐p300 + Flag‐XBP1s + L002 + MG132 groups and then treated with CHX for 0, 15, 30 and 60 min. B, The relative protein levels of XBP1s were analysed. ^*^ *P* <.05, the pcDNA3.1‐p300 + Flag‐XBP1s + L002 group vs the pcDNA3.1‐p300 + Flag‐XBP1s group. HEK293T cells were randomly assigned into the pcDNA3.1‐p300 + Flag‐XBP1s, pcDNA3.1 + Flag‐p300‐XBP1s + L002, pcDNA3.1‐p300 + Flag‐XBP1s + MG132, and pcDNA3.1‐p300 + Flag‐XBP1s + L002 + MG132 groups. C, After transfection with the indicated plasmids, HEK293T cells were immunoprecipitated with anti‐XBP1s antibody and immunoblotted with anti‐ubiquitin antibody. D, RAW264.7 cells were assigned to normal, hypoxia, hypoxia +0.1% DMSO, hypoxia +L002 and hypoxia +STF‐083010 groups. The Herpud1 protein levels were detected by Western blot. E, The relative protein level of Herpud1 was analysed. ^**^ *P* <.01 vs the normal group; ^##^ *P* <.01 vs the hypoxia group. F, The XBP1s‐binding sites on Herpud1 mRNA were shown. The unfolded protein response element (UPRE) TGACGTGG (10291‐19298) was located from −54 to −47. G, Luciferase reporter gene experiments were performed in HEK293T cells in the normal, Flag‐XBP1s + Luc‐Herpud1 WT, pcDNA3.1‐p300 + Flag‐XBP1 + Luc‐Herpud1 WT, Flag‐XBP1 + Luc‐Herpud1 MUT and pcDNA3.1‐p300 + Flag‐XBP1 + Luc‐Herpud1 MUT groups. Luciferase activity was analysed and normalized to Renilla luciferase activity. ^*^ *P* <.05, ^***^ *P* <.005 vs the normal group; ^##^ *P* <.01 vs the Flag‐XBP1s + Luc‐Herpud1 WT group. n = 4/group

How did p300 upregulate XBP1s expression? We performed a CHX chase assay in RAW264.7 cells with the proteasome inhibitor MG132, and the results clearly indicated that the p300 inhibitor L002 decreased the stability of XBP1s compared to that in the p300 + XBP1 co‐transfection group (Figure 3A,B), while MG132 reversed the change in the XBP1s protein level independent of L002. Accordingly, p300 inhibition increased the ubiquitination of XBP1s, while MG132 reversed the effect of p300 inhibition (Figure 3C). These findings suggested that p300, through its acetylase enzymatic activity, prevented the proteasomal degradation of XBP1s. Then, we focussed on Herpud1, an XBP1 target molecule. Western blot showed that hypoxia‐induced Herpud1 expression was inhibited by the p300 inhibitor L002 and the XBP1 splicing inhibitor STF‐083010 (Figure 3D,E). To confirm the direct binding between XBP1 and Herpud1 mRNA, the UPRE sequence TGACGTGG (10291‐19298), located at −54 to −47 relative to the beginning of the human Herpud1 promoter, was mutated and cloned into the Luc vector (Figure 3F). Next, XBP1 overexpression and Luc‐Herpud1 WT enhanced the transcriptional activity of the Herpud1 promoter compared to that in the normal group. Upon p300 overexpression, the transcriptional activity of the Herpud1 promoter further increased compared to that of the HA‐XBP1 + Luc‐Herpud1 WT group. However, Luc‐Herpud1 MUT displayed no enhanced transcriptional activity, neither with nor without p300 overexpression (Figure 3G). These data suggested that hypoxia‐induced p300 alleviated the proteasome‐dependent degradation of XBP1s and enhanced the transcriptional activity of XBP1s for Herpud1.

### 3.4 The hypoxia‐induced p300/XBP1s/Herpud1 axis facilitated RAW264.7 cell M2 polarization

**FIGURE 4:**
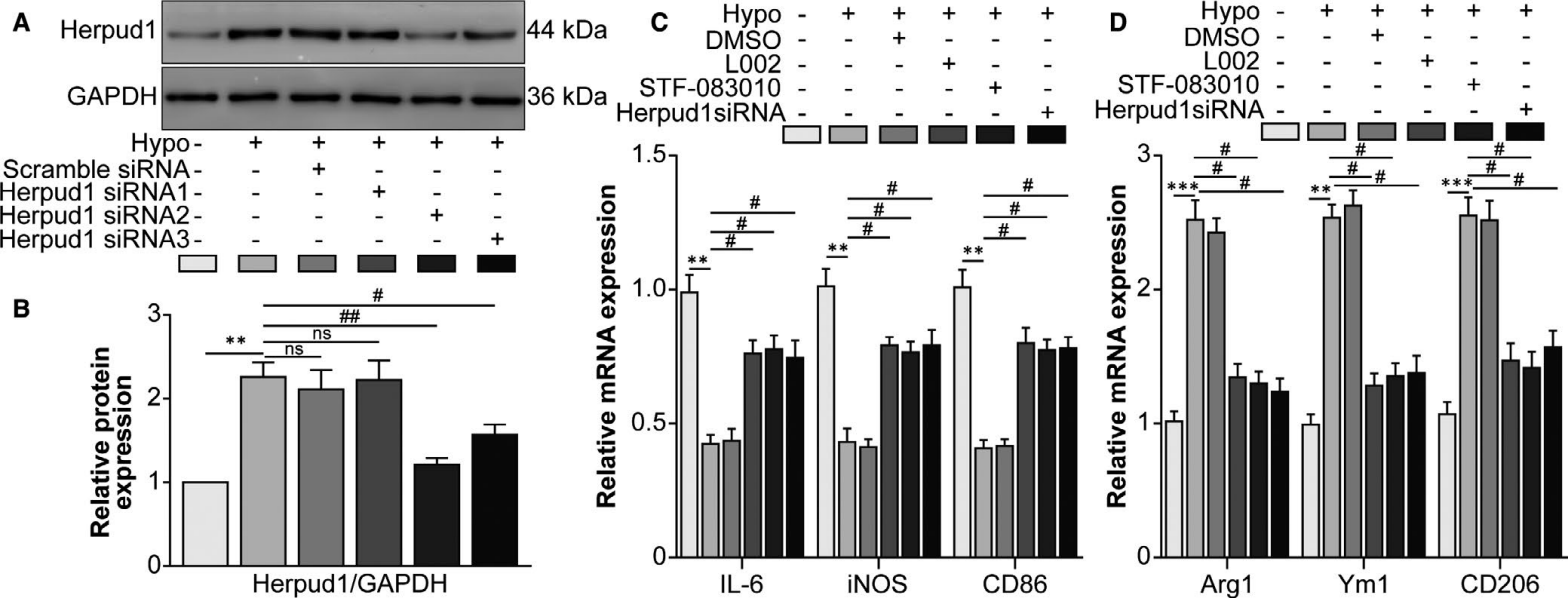
The hypoxia‐induced p300/XBP1s/Herpud1 axis facilitated RAW264.7 cell M2 polarization. A, Western blot was performed to detect Herpud1 protein levels in the normal, scrambled siRNA and Herpud1 siRNA transfection groups. B, Densitometric quantification showed the ratio of Herpud1 protein to GAPDH protein. ^**^ *P* <.01 vs the normal group; ^#^ *P* <.05, ^##^ *P* <.01 vs the hypoxia group. NS indicates no significant difference vs the hypoxia group. RAW264.7 cells were assigned to normal, hypoxia, hypoxia +0.1% DMSO, hypoxia +L002, hypoxia +STF‐083010 and hypoxia +Herpud1 siRNA groups. C, The mRNA levels of the M1‐type macrophage markers IL‐6, iNOS and CD86 were detected by qRT‐PCR. ^**^ *P* <.01 vs the normal group; ^#^ *P* <.05 vs the hypoxia group. D, The mRNA levels of the M2‐type macrophage markers Arg1 Ym1 and CD206 were detected by qRT‐PCR. ^**^ *P* <.01, ^***^ *P* <.005 vs the normal group; ^#^ *P* <.05 vs the hypoxia group. n = 4/group

Next, we wondered the effect of hypoxia‐induced p300/XBP1s/Herpud1 axis on the polarization of RAW264.7 cells. Following Herpud1 siRNA transfection, Herpud1 protein levels decreased, validating the efficiency of Herpud1 knockdown (Figure 4A,B). mRNA levels of the M1‐type macrophage markers IL‐6, iNOS and CD86 and the M2‐type macrophage markers Arg1, Ym1 and mannose receptor C‐Type 1 (CD206) were detected. Hypoxia decreased mRNA levels of the M1‐type markers, while p300 inhibition, XBP1 splicing inhibition and Herpud1 knockdown increased those of the M1‐type markers. Meanwhile, M2‐type and M1‐type macrophage markers showed the opposite tendencies (Figure 4C,D), suggesting that hypoxia‐induced p300/XBP1s/Herpud1 axis facilitated the M2 polarization of RAW2647 cells.

### 3.5 Knockdown of the p300/XBP1s/Herpud1 axis in RAW264.7 cells inhibited the proliferation, migration and tube formation of MCECs

**FIGURE 5:**
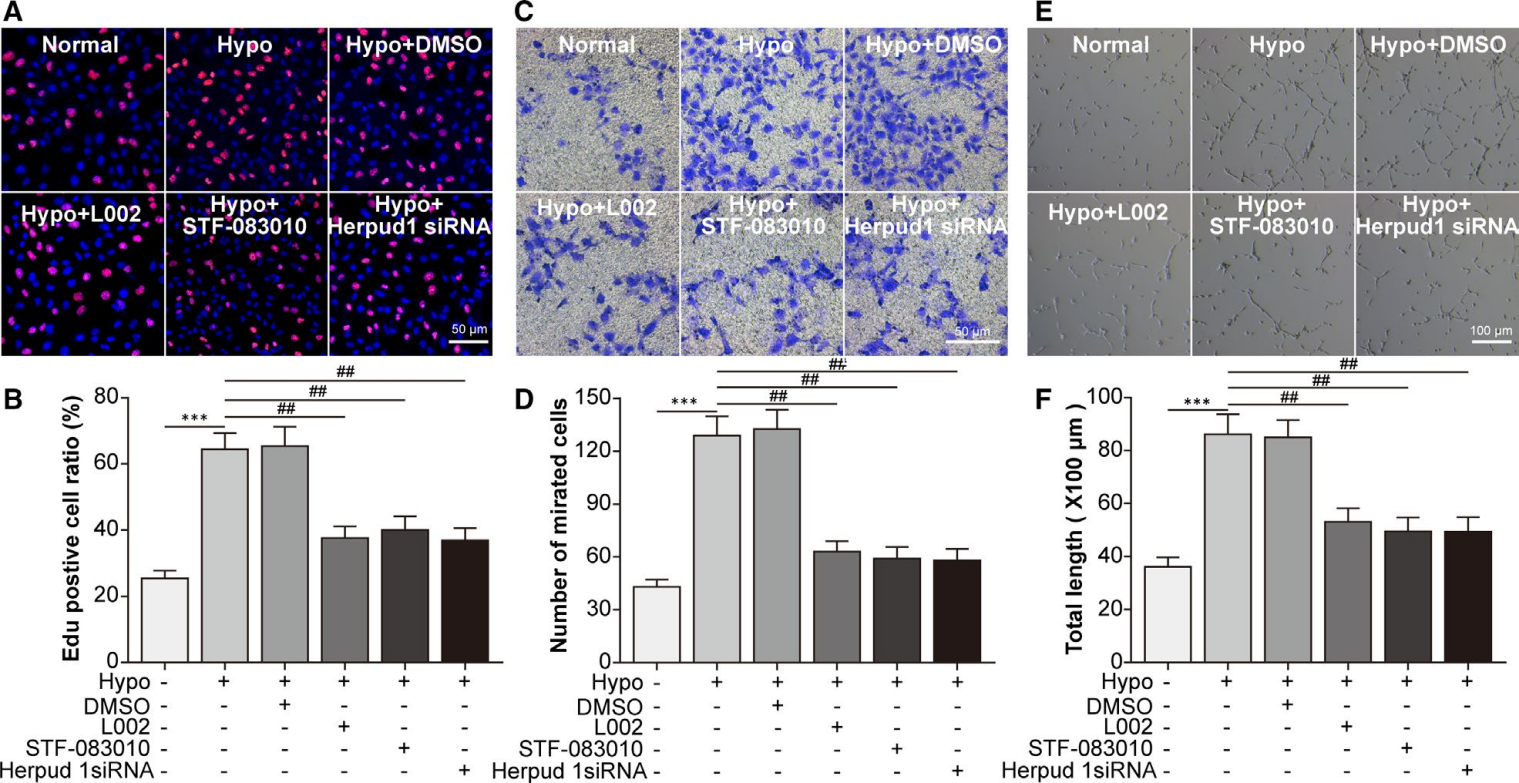
Knockdown of the p300/XBP1s/Herpud1 axis in RAW264.7 cells inhibited the proliferation, migration and tube formation of mouse choroidal endothelial cells. The CCM of RAW264.7 cells was collected to treat mouse choroidal endothelial cells (MCECs). RAW264.7 cells were assigned to normal, hypoxia, hypoxia +0.1% DMSO, hypoxia +L002, hypoxia +STF‐083010 and hypoxia +Herpud1 siRNA groups. A, EdU corporation assay was performed to detect the proliferation of MCECs. B, The mean ratio of the number of EdU‐positive cells vs the number of DAPI‐positive cells was analysed. C, Transwell assay was performed to detect the migration of MCECs. D, The mean number of migrated MCECs was analysed. E, Tube formation assay was performed, and representative results are shown in the panel. F, The total length of formed tubes was analysed. ^***^ *P* <.005 vs the normal group; ^##^ *P* <.01 vs the hypoxia group in Fig. 5B, 5D and 5F. Scale bar =50 μm in Fig. 6A and C; scale bar =100 μm in Fig. 6E. n = 4/group

What role did the p300/XBP1s/Herpud1 axis in RAW264.7 cells play in the behaviours of MCECs? The EdU incorporation assay showed that hypoxia‐treated conditioned culture medium (CCM) promoted the proliferation of MCECs, while p300 inhibition, XBP1 splicing inhibition and Herpud1 knockdown impaired the hypoxia‐induced proliferation of MCECs (Figure 5A,B). As shown by Transwell (Figure 5C,D) and tube formation (Figure 5E,F) assays, hypoxia‐treated CCM enhanced the migration and tube formation of MCECs, while p300 inhibition, XBP1 splicing inhibition and Herpud1 knockdown had the opposite effect. These results suggested that hypoxia‐induced p300/XBP1s/Herpud1 axis activation in RAW264.7 cells promoted the proliferation, migration and tube formation of MCECs.

### 3.6 The p300/XBP1s/Herpud1 axis increased in infiltrating M2‐type macrophages in mouse CNV lesions

**FIGURE 6:**
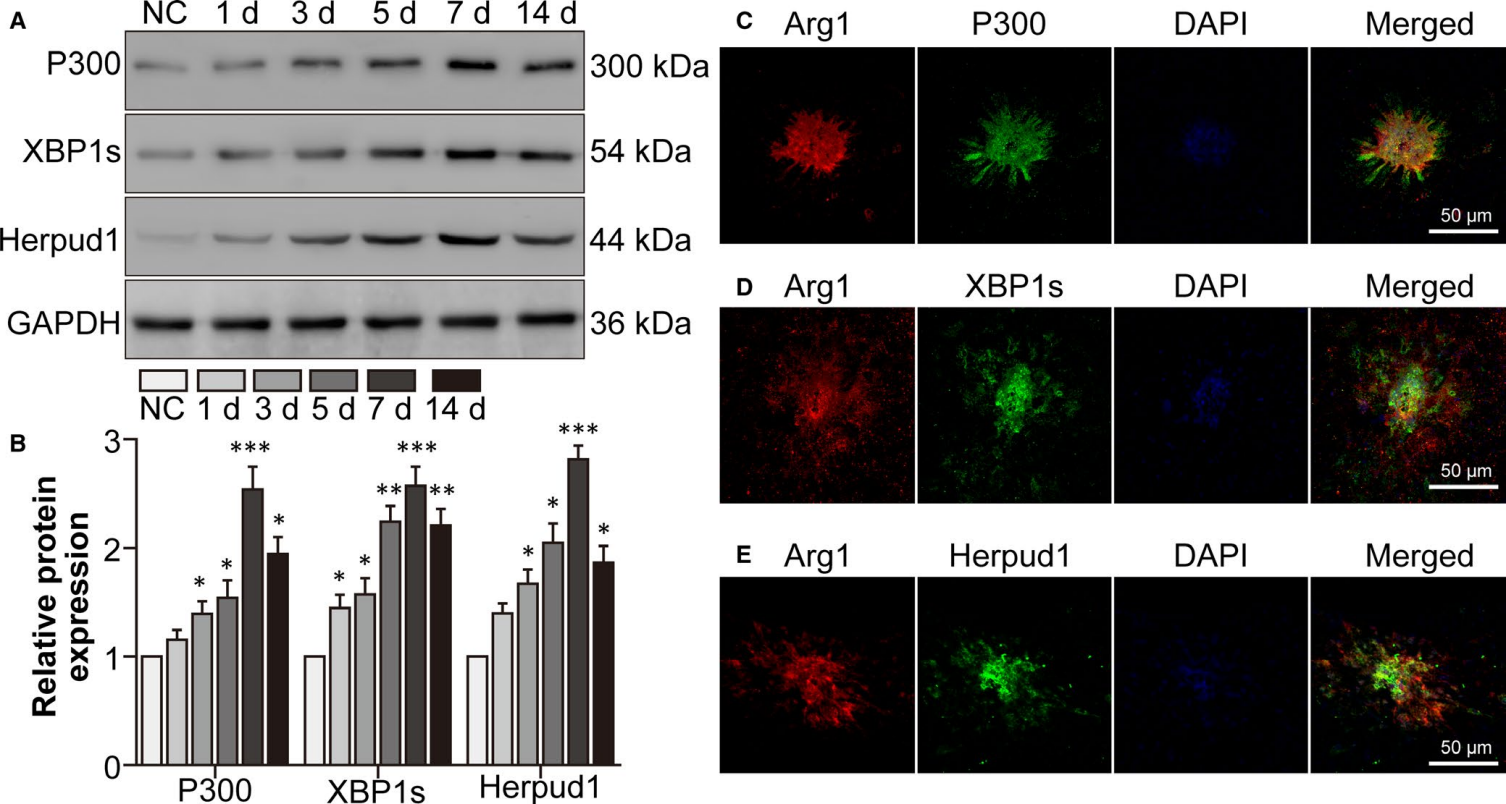
The P300/XBP1s/Herpud1 axis increased in infiltrating M2‐type macrophages in mouse CNV lesions. The mice were assigned to the normal control (NC), CNV 1 day, CNV 3 days, CNV 5 days, CNV 7 days and CNV 14 days groups. A, Western blot was performed to detect p300, XBP1s and Herpud1 protein levels in the mouse retina‐RPE‐choroid complexes. B, The relative protein levels of each molecule were analysed. ^*^ *P* <.05, ^**^ *P* <.01, ^***^ *P* <.005 vs the NC group. The mice were assigned into the NC and CNV 7 days groups. C, Double staining for Arg1 and p300 was performed with retina‐RPE‐choroid cryosections. D, Double staining for Arg1 and XBP1s was performed with retina‐RPE‐choroid cryosections. E, Double staining for Arg1 and Herpud1 was performed with retina‐RPE‐choroid cryosections. Scale bar =50 μm in Fig. 6C‐E. n = 4/group

Subsequently, the presence of the p300/XBP1s/Herpud1 axis was confirmed in a mouse laser‐induced CNV model. Western blot showed that p300, XBP1 and Herpud1 protein levels increased from the day following laser photocoagulation, peaked at day 7, and thereafter declined (Figure 6A,B). Additionally, Arg1 (an M2‐type macrophage marker)/p300 (Figure 6C), Arg1/XBP1 (Figure 6D) and Arg1/Herpud1 (Figure 6E) co‐localization inside the mouse retina‐RPE‐choroid complex increased following CNV. The data suggested that the p300/XBP1s/Herpud1 axis increased in infiltrating M2‐type macrophages in mouse CNV lesions.

### 3.7 Blockade of the p300/XBP1s/Herpud1 axis inhibited macrophage M2 polarization and alleviated CNV lesions

**FIGURE 7:**
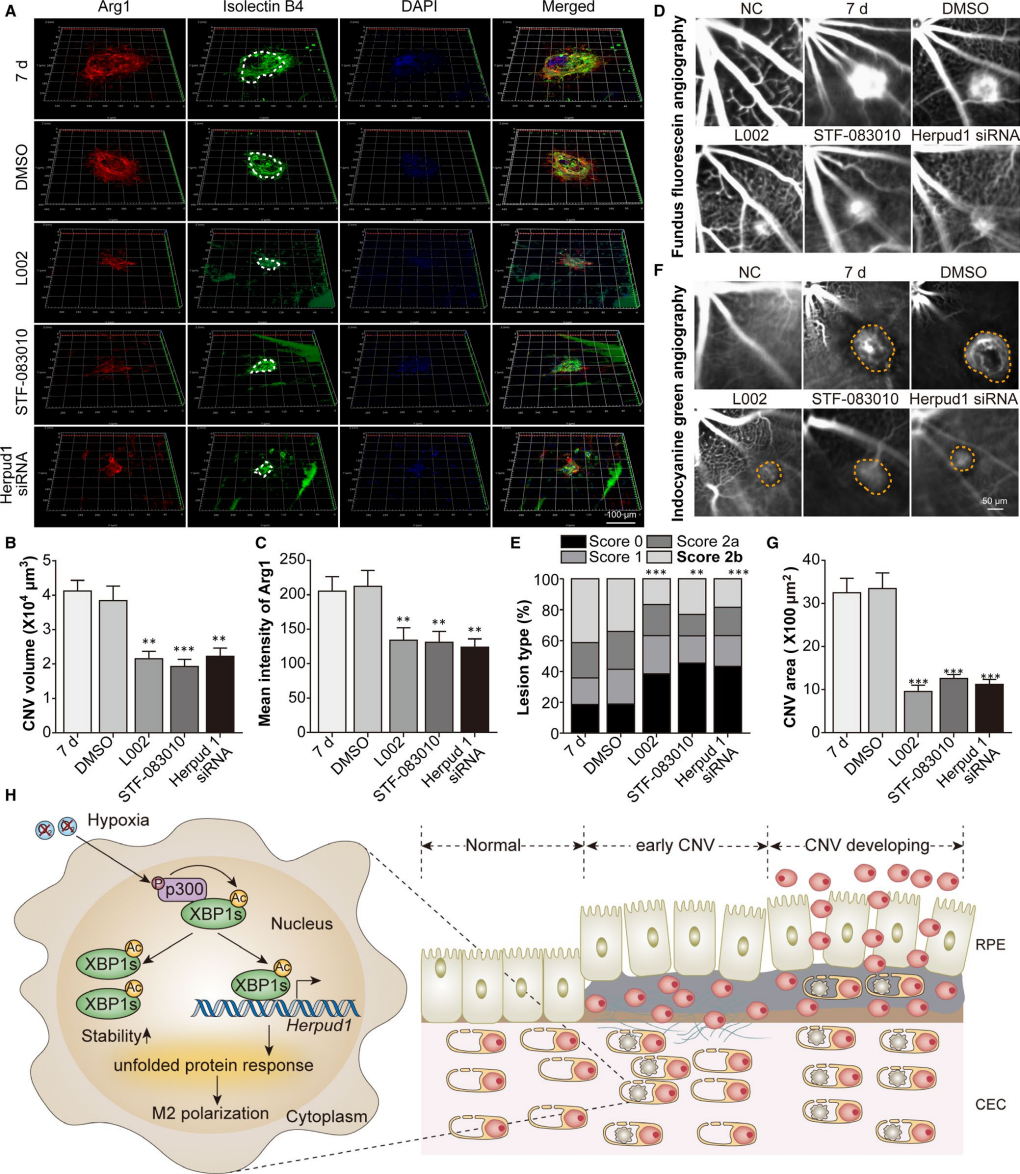
Blockade of the p300/XBP1s/Herpud1 axis inhibited macrophage M2 polarization and alleviated CNV lesions. Mice were assigned to the NC, CNV 7 days, CNV 7 days +0.1% DMSO, CNV 7 days +L002, CNV 7 days +STF‐083010 and CNV 7 days +Herpud1 siRNA groups. A, Double staining for Arg1 and IB4 was performed with choroidal flat mounts. B, The CNV volume derived from IB4 staining was analysed. ^**^ *P* <.01, ^***^ *P* <.005 vs the CNV 7 d group. C, The M2 polarization of macrophages represented by Arg1 staining was analysed. ^**^ *P* <.01 vs the CNV 7 d group. D, FFA was done to detect the leakage of CNV lesions. E, The leakage of CNV lesions was analysed. ^**^ *P* <.01, ^***^ *P* <.005 vs the CNV 7 d group. F, ICGA was done to detect the CNV area. G, The CNV area was analysed. ^***^ *P* <.005 vs the CNV 7 days group. n = 5/group. H, Schematic diagram of pathways was shown. Under hypoxic conditions, activated p300 interacted with XBP1s in infiltrating macrophages, causing the acetylation of XBP1, increasing the stability and transcription capability (Herpud1) of XBP1s, enhancing the UPR and promoting macrophage M2 polarization. M2‐type macrophages facilitated the proliferation, migration and tube formation of choroidal endothelial cells, thereby enhancing the development of CNV

Finally, we blocked the p300/XBP1/Herpud1 axis to explore its function in mouse CNV lesions. Double staining for Arg1 and IB4 (a vascular endothelial cell marker) showed that p300 inhibition, XBP1 splicing inhibition and Herpud1 knockdown alleviated M2‐type macrophage infiltration and decreased CNV volume (Figure 7A‐C). Moreover, FFA and ICGA showed that p300 inhibition, XBP1 splicing inhibition and Herpud1 knockdown alleviated laser‐induced CNV leakage (Figure 7D,E) and decreased the leakage area (Figure 7F,G), respectively. These data suggested that blockade of the p300/XBP1s/Herpud1 axis inhibited macrophage M2 polarization and alleviated CNV lesions.

## 4 DISCUSSION

The molecule p300 acts as a histone acetyltransferase. A previous study revealed that p300 increases the acetylation and protein stability and promotes the transcriptional activity of XBP1s.^15^ In this study, p300 protein levels were induced by hypoxia in RAW264.7 cells. Additionally, p300 enhanced the stability of XBP1s by interacting with XBP1s to inhibit the ubiquitination of XBP1s. Our results provide a novel mechanism for the effect of p300 for upregulating XBP1s expression and activity.

XBP1s, a mature form of XBP1, is an effector in the UPR and serves as a transcription factor. Simulation of the UPR by ER stress regulates the polarization of macrophages. For example, ER stress in macrophages derived from diabetic patients induced by exposure to either cholesterol or thapsigargin promoted the shift from the pro‐inflammatory M1 phenotype into the anti‐inflammatory M2 phenotype.^20^ However, macrophages isolated from the adipose tissue of CHOP‐knockout mice exhibited significantly increased numbers of the M2 subtype.^21^ These studies indicate that the polarization of macrophages might be controlled in a tissue‐specific manner as a response to the distinct pathology of host tissues. In our study, XBP1s directly transcribed Herpud1 to facilitate the M2 polarization of macrophages exposed to hypoxia, which proved the tissue‐specific manner of macrophage polarization responded to distinct stimulus.

M2 macrophages act as a kind of immunosuppressive and tumour‐promoting cell type and initiate debris scavenging, wound healing, tumorigenesis and angiogenesis. In addition, VEGF is mainly derived from infiltrating M2 macrophages, inducing neovascularization in AMD.^22^ Infiltrating macrophages interact with choroidal endothelial cells through Notch receptor 1 (Notch1) signalling during retinal sprouting angiogenesis.^23^ Therefore, M2 macrophage infiltration in CNV lesions is an emerging target for CNV therapy.^24^, ^25^ In our study, activation of the p300/XBP1s/Herpud1 axis in infiltrating M2 macrophages promoted the proliferation, migration and tube formation of choroidal endothelial cells. Furthermore, blockade of the p300/XBP1s/Herpud1 axis inhibited the M2 polarization of macrophages and alleviated the development of CNV, in consistent with previous studies.^8^, ^25^

Currently, p300 inhibitors have been applied in cellular and animal experiments for the treatment of diseases such as multiple cancers^26^ and fibrosis in the lung,^27^ kidney^28^ and heart.^29^ In our study, intraperitoneal injection of the p300 inhibitor L002 also ameliorated leakage and decreased the leakage area in laser‐induced CNV in mice. Except Herpud1 siRNA, L002 and STF‐083010 administrated via intraperitoneal injection is better than intravitreal injection to some extent, as it avoids ocular complications such as ocular hypertension and endophthalmitis.^30^

In general, the p300/XBP1s/Herpud1 axis in infiltrating macrophages increased the M2 polarization of macrophages and the development of CNV. However, there were several limitations in our study to be improved in the future, such as the absence of primary mouse macrophages and the side effects of L002, STF‐083010, and Herpud1 siRNA in mice.

## CONFLICTS OF INTEREST

All authors declare that there are no conflicts of interest.

## AUTHOR CONTRIBUTIONS

**Wendie Li:** Funding acquisition (equal); Investigation (equal); Methodology (equal); Project administration (equal); Resources (equal); Software (equal); Writing‐original draft (equal). **Ying Wang:** Conceptualization (equal); Data curation (equal); Investigation (equal); Writing‐original draft (equal). **Linling Zhu:** Formal analysis (equal); Funding acquisition (equal); Investigation (equal); Software (equal); Supervision (equal); Validation (equal); Visualization (equal). **Shu Du:** Funding acquisition (equal); Investigation (equal); Methodology (equal); Software (equal); Visualization (equal). **Jinghai Mao:** Data curation (equal); Formal analysis (equal); Software (equal). **Yanyan Wang:** Formal analysis (equal); Investigation (equal); Resources (equal); Software (equal); Validation (equal). **Sangsang Wang:** Data curation (equal); Formal analysis (equal); Software (equal). **Qingyun Bo:** Data curation (equal); Investigation (equal); Software (equal). **Yuanyuan Tu:** Data curation (equal); Formal analysis (equal); Investigation (equal); Writing‐original draft (equal); Writing‐review & editing (equal). **Quanyong Yi:** Funding acquisition (lead); Investigation (lead); Project administration (lead); Resources (equal); Visualization (equal); Writing‐original draft (equal); Writing‐review & editing (equal).

## Data Availability

The data that support the findings of this study are available from the corresponding author upon reasonable request.
